# A Hybrid Controller for a Soft Pneumatic Manipulator Based on Model Predictive Control and Iterative Learning Control

**DOI:** 10.3390/s23031272

**Published:** 2023-01-22

**Authors:** Yicheng Dai, Zhihao Deng, Xin Wang, Han Yuan

**Affiliations:** 1 School of Mechanical Engineering and Automation, Harbin Institute of Technology (Shenzhen), Shenzhen 518055, China; 2 Guangdong Key Laboratory of Intelligent Morphing Mechanisms and Adaptive Robotics, Shenzhen 518055, China

**Keywords:** pneumatic manipulator, dynamic modeling, hybrid controller

## Abstract

Due to the outstanding characteristics of the large structural flexibility and strong dexterity of soft robots, they have attracted great attention. However, the dynamic modeling and precise control of soft robots face huge challenges. Traditional model-based and model-free control methods find it difficult to obtain a balance between complexity and accuracy. In this paper, a dynamic model of a three-chamber continuous pneumatic manipulator is established based on the modal method. Moreover, a hybrid controller integrating model predictive control (MPC) and iterative learning control (ILC) is proposed, which can simultaneously perform model parameter learning and trajectory tracking control. Experimental results show that the proposed control method can optimize the parameters of the dynamic model in real time with less iterations than the traditional model-free method and have good control performance in trajectory tracking experiments. In the future, the proposed dynamic model and the hybrid controller should be verified on a multi-section manipulator.

## 1. Introduction

Differently from rigid robots, soft continuum manipulators benefit from great compliance, lightweight, safer interaction, and a high power-to-weight ratio [1,2,3]. There has been increasing research on continuum robotics in recent years. They have a promising future and provide new capabilities in some fields, such as surgical applications [4,5] where continuum robots perform better when passing through narrow passages between organs for their flexible mechanisms and dexterous mobility [6]. There have been many attempts at soft robots in bionics design, such as octopus tentacles [7,8], manta [9], caterpillars [10], and elephant trunks [11,12]. Soft continuum manipulators are mainly divided into two categories according to their actuation methods: cable-driven manipulators [13,14] and pneumatically actuated manipulators [15,16]. Compared with general cable-driven continuum robots, pneumatic robots are more lightweight and compliant. This is due to the physical properties of the air used for both actuation and structure. However, the advantages of soft pneumatic continuum robots come with the trade-off of complexity in their modeling and control [17]. Due to the continuity, non-linear flexible material, and the unlimited number of degrees of freedom of the manipulator [18], it is challenging to establish its model.

The common assumptions of traditional rigid multi-segment robots do not apply to soft robots. At this stage, for soft manipulators, kinematics modeling is mainly studied [19,20]. OctArm by Walker et al. [21] is a successful implementation of a multi-section continuum robot using a pneumatic muscle actuator, according to the model proposed by Jones et al. [22]. However, the system dynamics is still a challenge and the using of different materials will make it even more difficult. Soft materials such as silicone, eco flex, rubber, or gel have high hyper-elasticity [23], which needs to be considered in the continuum mechanics model instead of extending the classical elastic beam theory used for rigid robots to soft robots. Early dynamic model derivations are mainly presented by Chirikjian [24] for a hyper-redundant manipulator, by Khalil et al. [25] for a serial eel-like robot, and by Matsuno and Sato for a snake robot [26]. However, most robots in these studies have rigid connections. They are not suitable for robots with completely flexible structures. Tatlicioglu et al. [27] deduced the dynamic model of the continuous manipulator arm. Godage I et al. [28] further deduced the dynamic model of each cavity in the three-dimensional space, but his study was limited in special experimental materials and background. The latest study on the dynamic model of soft robots is based on the Cosserat rod model proposed by Gazzola et al. [29] to capture the shearing, bending, and stretching of the continuum section. Although this model has extensively proven its applicability in various environments, its calculation process is too complicated to be applied in real-time control. In terms of the control algorithm of soft robots, the existing control methods are mainly divided into three categories: model-free method, model-based method, and the method based on hybrid controller [30]. Model-free methods are usually based on machine learning techniques or empirical evidence. Although such controllers have great advantages in highly nonlinear and non-uniform conditions, stability and convergence are difficult to guarantee due to probabilistic or non-parametric properties [31].

In applications that require high stability and predictable system behavior, model-based methods are a more appropriate choice. Model-based controllers usually require analytical models to derive the controller, and these controllers tend to perform better in terms of accuracy and reliability [32]. However, they need to define an accurate dynamic model of the soft robot which is not easy due to its flexible structure [33]. In addition, advanced accurate modeling methods are limited due to their computational complexity and the cost of the required sensors. Hybrid controllers [30] combine model-free and model-based controllers, usually based on the model to capture the main part of the system’s inherent properties, which are used to learn the model, thereby supplementing the dynamic uncertainty.

In this article, we use the modal method based on the CP kinematics of the continuous soft robot. It reduces the degree of numerical nonlinearity and eliminates singularity problems effectively, speeding up the solution of the dynamic model. The dynamic model of the three-chamber soft manipulator presented in this paper was established by this method. Based on the derived dynamic model, MPC and ILC are combined as a hybrid controller, which can perform model parameter learning and trajectory tracking at the same time. The simulation result shows that the control algorithm proposed in this paper can optimize and update the model parameters in real time. Compared with the traditional model-free closed-loop feedback control (e.g., PID controller), it can obtain higher tracking accuracy with fewer iterations. In addition, it can obtain more accurate model parameters according to different prototypes, and adapt to the inconsistent prototype parameters caused by manufacturing process.

Compared with existing research, the major contribution of this paper is as follows.

1. Taylor expansion was used to solve kinematics singular solution problems.
2. Universal dynamic modeling for the three-chamber continuous pneumatic manipulator is presented based on the modal method.
3. A hybrid controller integrating the model predictive control method and iterative learning control method was proposed.

**Figure 1:**
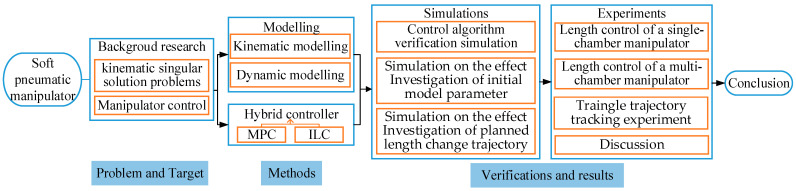
The scientific flowchart of this paper.

The scientific flowchart of this paper is shown in Figure 1. The rest of this paper is organized as follows. Section 2 describes the designing and manufacturing method of the multi-chamber continuous soft manipulator. Section 3 describes the kinematics and dynamic model. Section 4 describes the derivation process of the model-based parameter adaptive learning control algorithm. In Section 5, simulations based on different control algorithms are conducted and compared. In Section 6, experiments are carried out and the results are analyzed. Finally, conclusions are made in Section 7.

## 2. System Description

**Figure 2:**
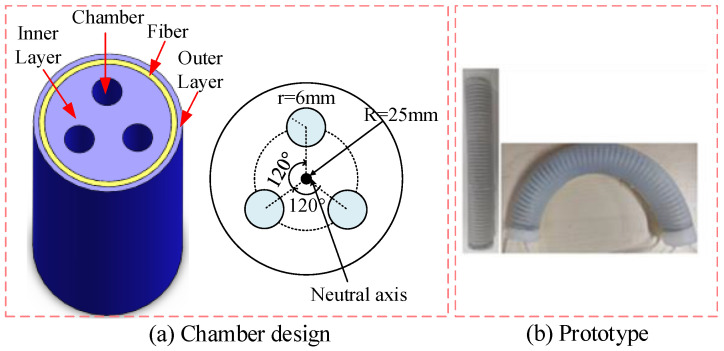
Structure of the soft continuum manipulator.

The prototype is shown in Figure 2a. It contains chambers, inner layer, fiber, and the outer layer. The three chambers are separated by 120° intervals and their radii is equal. This structure enables the soft pneumatic manipulator to extend and contract, as well as bend in the space. Inspired by the design in [34], each pneumatic chamber is reinforced with a thin nylon fiber, forming a tight spiral on the outside of the cavity in the form of a diagonal. The radial expansion of the soft manipulator is limited and axial stretching is allowed. The purpose of covering the fiber layer with a silica layer is to prevent the fiber from slipping, which will affect the bending angle of the manipulator.

**Figure 3:**
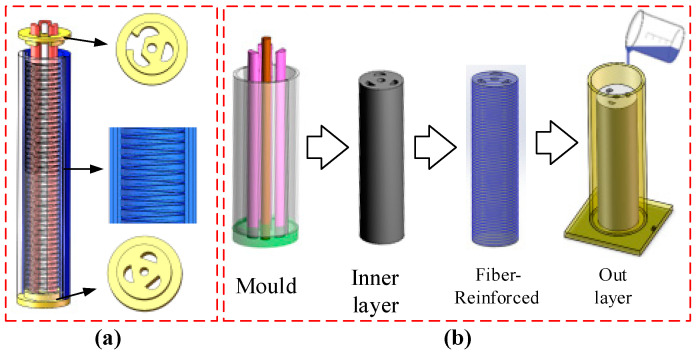
The manufacturing process of the multi-chamber soft pneumatic manipulator. (**a**) Design of the inner layer mold. It is composed of 3 chambers with a diameter of 6mm and an inner wall with a diameter of 25 mm. The inner wall is spirally convex, which is convenient for subsequent Fiber winding. The top fixing is used for sealing and positioning and the mold is made by 3D printing. (**b**) The manufacturing process of the soft manipulator, including the inner and outer mold design.

The manufacturing process of the soft pneumatic manipulator is shown in Figure 3. Firstly, silica gel (Ecoflex-0300) is used as the raw material, and the A-liquid and the B-liquid are mixed with the same quality. After stirring, they are put into a vacuum box to remove the dissolved air so as to avoid bubbles forming in the soft arm. Subsequently, the inner molds, as shown in Figure 3a, were coated with a release agent (Vaseline) to facilitate demolding. The silica gel is poured into the mold and placed in the heating box for solidification. After the manipulator is formed, the fiber layer is wound around it. The thickness of the fiber layer is 2 mm. Then it is put into the outer layer mold again, and the evenly mixed silica gel is poured.

## 3. System Modeling

### 3.1. Kinematic Modeling

**Figure 4:**
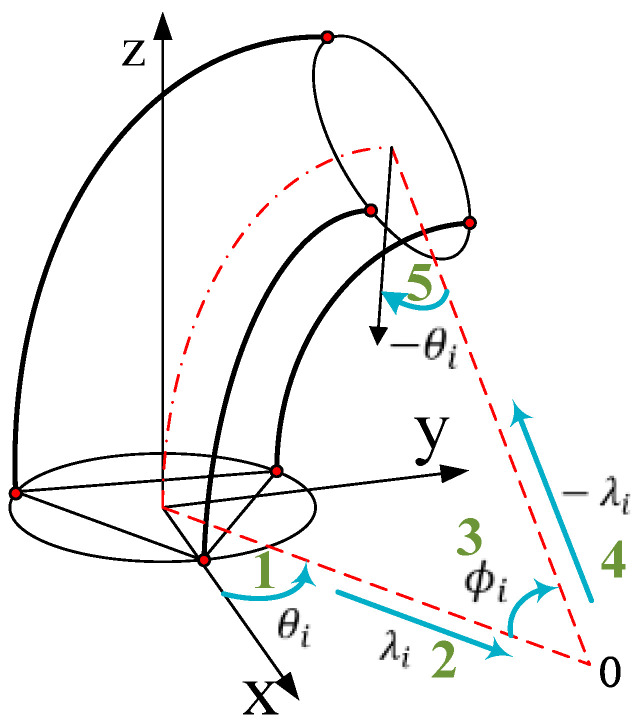
Schematic of the kinematic parameters of the proposed soft pneumatic manipulator.

Based on the assumption of constant curvature, a continuous curved section can be described by three independent parameters {θ,φ,λ}. As shown in Figure 4, *θ* indicates the rotating angle, which is the angle between the bending plane and the *x*-axis. *φ* indicates the bending angle. λ indicates the bending radius. Note the length variation of the three chambers $\left\{l_{1},l_{2},l_{3}\right\}$, the original length $L_{0}$ and the radius r of soft manipulator. The parameter relationship of the bending section can be written as:

(1) $$\lambda =\frac{\left(3L_{0}+l_{1}+l_{2}+l_{3}\right)r}{2\sqrt{l_{1}{}^{2}+l_{2}{}^{2}+l_{3}{}^{2}-l_{1}l_{2}-l_{1}l_{3}-l_{2}l_{3}}}$$

(2) $$\phi =2\sqrt{l_{1}{}^{2}+l_{2}{}^{2}+l_{3}{}^{2}-l_{1}l_{2}-l_{1}l_{3}-l_{2}l_{3}}\;/(3R)$$

(3) $$\theta =\tan^{-1}\left(\sqrt{3}\left(l_{3}-l_{2}\right)/(l_{2}+l_{3}-2l_{1})\right)$$

As for soft pneumatic manipulators, robot mapping describes the coordinate transformation between task space and configuration space. While the traditional Denavit–Hartenberg [35] is not applicable due to the flexibility of the proposed manipulator, the homogeneous transformation matrix can be derived by applying the standard rotational and translational matrices. As shown in Figure 4, according to the bending parameters {θ,φ,λ}, the homogeneous transformation matrix shows the relationship between the base coordinate system and the tip end coordinate system of the manipulator. It can be written as:

(4) $$T^{c}\left(\xi ,q\right)=R_{z}\left(\theta \right)P_{x}\left(\lambda \right)R_{y}\left(\xi \phi \right)P_{x}\left(-\lambda \right)R_{z}^{T}\left(\theta \right)=\left[\begin{array}{cc} R^{c}\left(\xi ,q\right) & P^{c}\left(\xi ,q\right)\\ 0_{1\times 3} & 1 \end{array}\right]$$

where ξ∈[0,1] represents the normalized position parameter. When located at the base, ξ=0. When located at the top, ξ=1. The generalized coordinates $q={\left[l_{1}\;l_{2}\;l_{3}\right]}^{T}$. ***R**_z_* and ***R**_Y_* are the rotation matrices about the *z*-axis and *y*-axis, respectively. ***P**_x_* is the homogeneous translation matrix. Some elements (see Appendix A for details) are as follows:

(5) $$\begin{array}{l} {\left[R^{c}\right]}_{11}=\cos\left(\xi \phi \right)\cos^{2}\theta +\sin^{2}\theta \\ {\left[R^{c}\right]}_{12}=\cos\theta \sin\theta \left(\cos\left(\xi \phi \right)-1\right)\\ {\left[R^{c}\right]}_{13}=\sin\left(\xi \phi \right)\cos\theta \\ {\left[P^{c}\right]}_{1}\;=\lambda \cos\theta \left(1-\cos\left(\xi \phi \right)\right) \end{array}$$

If the pressure in the three chambers is the same, the manipulator will stretch along the axial direction. At this time, both the bending angle and the bending radius tend to infinity, which leads to singularities in the kinematics solution. On the other hand, from the above Equations (1)–(3), it can be seen that bringing $\left\{l_{1},l_{2},l_{3}\right\}$ into {θ,φ,λ}, respectively, and then the elements in the homogeneous transformation matrix, will produce many triangular and denominator terms. As a result, the calculation will be very complicated. Thus, in this paper, the high-order Taylor expansion of each element will be expressed as a numerically stable multivariate polynomial form. The modal matrix can be obtained as follows:

(6) $$\begin{array}{l} {\left[R\right]}_{11}={\left(l_{2}-2l_{1}+l_{3}\right)}^{2}\left(\frac{-\xi^{2}}{18r^{2}}+\frac{-\mu^{2}\xi^{4}}{486r^{4}}+\frac{-\mu^{4}\xi^{6}}{32805r^{6}}\right)+1\\ {\left[R\right]}_{12}=-\sqrt{3}\left(l_{2}-l_{3}\right)\left(l_{2}-2l_{1}+l_{3}\right)\left[\frac{-\xi^{2}}{18r^{2}}+\frac{u^{2}\xi^{4}}{486r^{4}}-\frac{u^{4}\xi^{6}}{32805r^{6}}\right]\\ {\left[R\right]}_{13}=\left(l_{2}-2l_{1}+l_{3}\right)\left[\frac{\xi }{3r}-\frac{2u^{2}\xi^{3}}{81r^{3}}+\frac{2u^{4}\xi^{5}}{3645r^{5}}\right]\\ {\left[P\right]}_{1}=-\left(3L_{0}+l_{1}+l_{2}+l_{3}\right)\left(l_{2}-2l_{1}+l_{3}\right)\left[\frac{-\xi^{2}}{18r}+\frac{u^{2}\xi^{4}}{486r^{3}}-\frac{u^{4}\xi^{6}}{32805r^{5}}\right] \end{array}$$

where $u=\sqrt{l_{1}^{2}+l_{2}^{2}+l_{3}^{2}-l_{1}l_{2}-l_{1}l_{3}-l_{2}l_{3}}$. It can be found that this model is completely composed of numerically stable multivariate polynomials, which eliminates the problem of singularities and reduces the complexity of calculations, making it easy to find derivation.

### 3.2. Dynamic Modeling

When establishing the dynamic model, the curved section is divided into countless small slices. The thickness of each slice is s⋅Δξ, where s=λφ represents the arc length of the central axis. Note the modal rotation matrix as $R\left(q,\xi \right)\in {\mathbb{R}}^{3\times 3}$ and the modal position vector as $P\left(q,\xi \right)\in {\mathbb{R}}^{3}$.

The linear velocity at the center of a slice is:

(7) $$v_{\xi }^{b}=R^{T}\cdot \dot{P}\left(q,\xi \right)=R^{T}\frac{\partial P\left(q,\xi \right)}{\partial q^{T}}\dot{q}$$

and the angular velocity with respective to the base coordinate axes is:

(8) $$w_{\xi }^{b}=R^{T}{\left(\dot{R}R^{T}\right)}^{\vee }={\left(R^{T}\dot{R}\right)}^{\vee }$$

Thus, the velocity vector of a single slice is:

(9) $$\delta M\left(q\right)=\delta m\cdot diag\left\{1,1,1,\frac{r^{2}}{4},\frac{r^{2}}{4},\frac{r^{2}}{4}\right\}$$

where $J_{\xi }^{b}\left(q\right)$ is the modal Jacobian matrix.

Note the mass of continuum section is m, the inertial matrix of any slice is:

(10) $$\delta M\left(q\right)=\delta m\cdot diag\left\{1,1,1,\frac{r^{2}}{4},\frac{r^{2}}{4},\frac{r^{2}}{4}\right\}$$

According to the velocity vector, the kinetic energy of any slice can be derived by:

(11) $$\delta K\left(q,\dot{q}\right)=\frac{1}{2}{\left(V_{\xi }^{b}\right)}^{T}\cdot \delta M\left(q\right)\cdot \left(V_{\xi }^{b}\right)=\frac{1}{2}{\left(J_{\xi }^{b}\dot{q}\right)}^{T}\cdot \delta M\left(q\right)\cdot \left(J_{\xi }^{b}\dot{q}\right)$$

and with the assumption of equal density, the total kinetic energy is then calculated by integrating all slice kinetic energies as:

(12) $$K\left(q,\dot{q}\right)=\int_{0}^{1}\delta K\left(q,\dot{q}\right)d\xi =\frac{1}{2}{\dot{q}}^{T}\cdot M\left(q\right)\cdot \dot{q}$$

where the generalized mass matrix is defined as in Equation (13)

(13) $$M\left(q\right)=\int_{0}^{1}{\left(J_{\xi }^{b}\right)}^{T}\cdot \delta M\left(q\right)\cdot \left(J_{\xi }^{b}\right)d\xi$$

Due to the particularity of the silicone material, the potential energy of the soft manipulator consists of three parts: gravitational potential energy, elastic potential energy, bending potential energy. The gravitational potential energy can be calculated by modal position vector as:

(14) $$P_{G}\left(q\right)=m\int_{0}^{1}P\left(q,\xi \right)\cdot gd\xi$$

The elastic potential energy is caused by the elongation or contraction of the silicone material chamber. It can be described as:

(15) $$P_{E}\left(q\right)=\frac{1}{2}q^{T}\cdot K_{e}\cdot q$$

where $K_{e}=diag\left\{k_{e1},k_{e2},k_{e3}\right\}$ is the elastic stiffness matrix of the joint space and $k_{ei}$ is the elastic stiffness coefficient of each chamber.

To simplify the calculation of the bending potential energy, the assumption is cited that the bending potential energy is proportional to the square of the bending angle. Thus, the bending potential energy of soft continuum manipulator can be derived by

(16) $$P_{B}\left(q\right)=\frac{1}{2}K_{b}{\left(\frac{\phi }{2}\right)}^{2}=K_{b}\left(\frac{l_{1}{}^{2}+l_{2}{}^{2}+l_{3}{}^{2}-l_{1}l_{2}-l_{2}l_{3}-l_{1}l_{3}}{18R^{2}}\right)$$

where $K_{b}$ is the bending stiffness coefficient of the arm which is assumed to be constant. In summary, the potential energy of the soft continuum manipulator is

(17) $$\;P\left(q\right)=P_{G}\left(q\right)+P_{E}\left(q\right)+P_{B}\left(q\right)$$

The Lagrangian function of the system is defined by ***L*** = ***K*** − ***P***, and the equation of motion can be obtained by applying the Euler–Lagrange equation formulated in Equation (18).

(18) $$\frac{d}{dt}\frac{\partial L}{\partial \dot{q}}-\frac{\partial L}{\partial q}=Pa$$

The newly derived dynamics of the soft pneumatic manipulator is obtained in matrix form as:

(19) $$M\left(q\right)\ddot{q}+C\left(q,\dot{q}\right)\dot{q}+G\left(q\right)=F$$

where $C\left(q,\dot{q}\right)$ is the centrifugal and Coriolis forces matrix that can be derived by:

(20) $$c_{kj}=\sum_{i=1}^{3}\frac{1}{2}\left[\frac{\partial M_{kj}}{\partial q_{i}}+\frac{\partial M_{ki}}{\partial q_{j}}-\frac{\partial M_{ij}}{\partial q_{k}}\right]\cdot {\dot{q}}_{i}$$

and G(q) is the conservative forces vector that can be derived by $G(q)=\partial p/\partial q_{i}$.

## 4. Control Algorithm

**Figure 5:**
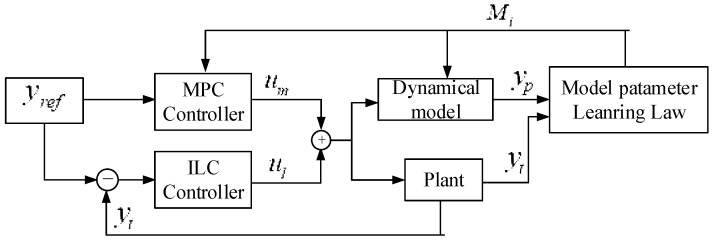
Parameter-adaptive-learning control diagram based on the soft continuum section dynamic model. Model predictive control (MPC) and iterative learning control (ILC), which are used as feedback and feedforward, are combined to iteratively learn model parameters, respectively.

In the process of establishing the mathematical model, in order to simplify the modeling difficulty, assumptions such as the constant curvature [36] and equal linear density were made. However, due to the non-linearity of the soft material itself, the relationship between the pressure and the length change of the chambers based on the dynamic model is not completely accurate. In order to further improve the accuracy of the model, a parameter adaptive learning control method based on the dynamic model is proposed. The control algorithm diagram is shown in Figure 5 as follows.

### 4.1. Parameters of the Control Model

According to the dynamic model of the continuous section, the motion equation of a single chamber after decoupling can be equivalently expressed as:

(21) $$J\ddot{q}+D\dot{q}+Kq=aP$$

where J is the equivalent moment of inertia, D is the equivalent damping coefficient, K is the equivalent stiffness, a is the cross-sectional area. Converted to the form of the state space equation:

(22) $$\dot{x}=Ax+Bu;\;y=Cx$$

Through the Laplace transform, the system transfer function is obtained as:

(23) $$G\left(s\right)=\frac{Y\left(s\right)}{U\left(s\right)}=C{\left[sI-A\right]}^{-1}B=\frac{a}{J}/(s^{2}+Ds+\frac{K}{J})$$

However, in a real control situation, the system runs discretely. In this paper, a zero-order holder is used to discretize the continuous system, and the sampling time is set as 0.1 s. The system pulse transfer function is derived through *z* transformation:

(24) $$G\left(z\right)=(p_{1}z^{-1}+p_{2}z^{-2})/(1+p_{3}z^{-1}+p_{4}z^{-2})$$

Thus, the control model parameters can be expressed as $p={\left[\begin{array}{cccc} p_{1} & p_{2} & p_{3} & p_{4} \end{array}\right]}^{T}$.

### 4.2. Hybrid Controller

The soft pneumatic manipulator is assumed to be a linear time-invariant system. A linear MPC controller will be used. The cost function of the model predictive control method is:

(25) $$\underset{u_{i}}{min}\sum_{k=1}^{k=H}\left\{Q{\left(y_{i}^{ref}\left(k\right)-y_{i}\left(k\right)\right)}^{2}+Ru_{i}^{2}\left(k\right)\right\}$$

Subject to:

(26) $$\begin{array}{l} x_{i}\left(k+1\right)=Ax_{i}\left(k\right)+Bu_{i}\left(k\right);\;y_{i}\left(k\right)=Cx_{i}\left(k\right)\\ y_{min}\leq y_{i}\left(k\right)\leq y_{max};\;u_{min}\leq u_{i}\left(k\right)\leq u_{max} \end{array}$$

where $y_{ref}$ is the reference length of the chamber, Q and R are the output and input weight factors, respectively, H is the prediction horizon. The input pressure of the soft pneumatic manipulator corresponding to the system parameters at the sampling time is optimized through model predictive control. The optimized input air pressure is used as the feedback pressure.

Iterative learning control has no requirements on the model, but the dynamic model parameters of the soft pneumatic manipulator derived previously can be used as the starting point of the iteration, which can greatly accelerate the iteration speed and reduce the number of iterations.

(27) $$\begin{array}{l} u_{i+1}\left(k\right)=u_{i}\left(k\right)+\lambda E_{i}^{r}\left(k+1\right)\\ y_{i}^{ref}={\left[\begin{array}{cccc} y_{i}^{ref}\left(1\right) & y_{i}^{ref}\left(2\right) & \cdots & y_{i}^{ref}\left(N\right) \end{array}\right]}_{N\times 1}^{T}\\ y_{i}={\left[\begin{array}{cccc} y_{i}\left(1\right) & y_{i}\left(2\right) & \cdots & y_{i}\left(N\right) \end{array}\right]}_{N\times 1}^{T} \end{array}$$

where $E_{i}^{r}=y_{i}^{ref}-y_{i}$ representing error between reference length and actual chamber length; *k* is sampling instant; *i* is the number of iterations. The feedforward input coefficient *λ* is a constant.

### 4.3. Model Parameters Iterative Learning Law

The relationship between model input and output is expressed as:

(28) $$\overset{⌢}{y}\left(kt\right)=\frac{p_{i}^{1}z^{-1}+p_{i}^{2}z^{-2}}{1+p_{i}^{3}z^{-1}+p_{i}^{4}z^{-2}}u\left(kt\right)$$

where $\overset{⌢}{y}\left(kt\right)$ is the model estimated chamber length. Through the inverse *z* transformation, we can obtain:

(29) $$\varphi \left(k\right)={\left[\begin{array}{cccc} u\left(k-1\right) & u\left(k-2\right) & -\overset{⌢}{y}\left(k-1\right) & -\overset{⌢}{y}\left(k-2\right) \end{array}\right]}^{T}$$

where *k* = 2, 3, …, *N*, and $N=T/T_{s}$, $T_{s}$ is sampling time. Then we have:

(30) $$\overset{⌢}{y}\left(k\right)=\varphi {\left(k\right)}^{T}p$$

Input the same pressure, the error between the actual change of chamber length and the change predicted by the model is:

(31) $$E_{i}^{t}=y\left(k\right)-\overset{⌢}{y}\left(k\right)$$

where $\overset{⌢}{y}\left(k\right)=\varphi {\left(k\right)}^{T}p_{i}$ is the estimated length change, $y\left(k\right)=\varphi {\left(k\right)}^{T}p_{t}$ is the true length change and $p_{t}$ is the true model parameter.

The estimated model is constantly being updated by minimizing the error $E_{i}^{t}$, as shown by the following quadratic function. Then the Gauss–Newton method [37] is applied to update the model parameters.

(32) $$\underset{\theta_{i}}{min}E_{i}^{t}{}_{2}^{2}$$

### 4.4. Parameter-Adaptive-Learning Control Algorithm

A complete algorithm description about parameter-adaptive-learning control based on the soft continuum section dynamic model is presented in Algorithm 1. Note $\varepsilon_{r}$ as the error tolerance between the reference length and the actual length. $\varepsilon_{t}$ is the error tolerance between the actual length and the model estimated length.
**Algorithm 1.** Model parameters learning adaptive control
Step1: Initialize the model parameter $M_{0}$, Plan length trajectory $y_{ref}$;
Step2: Obtain the initial pressure $u_{0}$ based on MPC;Step3: Compare with length tolerance $\varepsilon_{t}$ and $\varepsilon_{r}$.Step4: Obtain $u_{i}^{m}$ trough model parameters iterative learning law and MPCStep5: Obtain $u_{i}^{l}$ based trough ILCStep6: Obtain total input pressure $u_{i}$, real chamber length $y_{i}^{t}$ and model estimated length $y_{i}^{p}$ trough real system and estimated model, respectively. Then go to step 3.

To summarize, in this section, a model-based parameter adaptive learning control algorithm is proposed for the soft pneumatic manipulator. The previously established dynamics model of the manipulator is used. A hybrid controller integrating MPC and ILC is proposed and integrated into the parameter learning control law. Real-time optimization and update of model parameters is realized in the form of online learning. It can improve the accuracy of the dynamic model and achieve trajectory tracking with a small number of iterations. This control algorithm is verified by simulation, and results show that the control proposed algorithm has faster convergence and higher control precision.

## 5. Simulation

In order to verify the effect of the control algorithm on the parameter learning of the dynamic model and the desired chamber length change trajectory tracking, a single-chamber soft pneumatic manipulator is used to carried out the verification simulation.

### 5.1. Control Algorithm Simulation

The dynamic parameters of the soft continuous section are shown in Table 1.

Then we can obtain the initial continuous time model:

(33) $$G\left(s\right)=4/(s^{2}+1.78s+173)$$

The discretization of the control system is realized by using the zero-order holder with the sampling time of 0.1 s. The discretization result is:

(34) $$G{\left(z\right)}_{m}=\frac{0.016z^{-1}+0.015z^{-2}}{1-0.468z^{-1}+0.837z^{-2}}$$

and the initial parameter of the model is $p_{init}={\left[0.016\;0.015\;\;-0.0468\;0.837\right]}^{T}$. In the simulation, we assume that the real continuous-time system is:

(35) $$G{\left(s\right)}_{t}=8/(s^{2}+4s+400)$$

Similarly, the discretization result is:

(36) $$G{\left(z\right)}_{t}=\frac{0.025z^{-1}+0.021z^{-2}}{1+0.666z^{-1}+0.671z^{-2}}$$

The reference length which represents the change of the chamber length is set to be the tracking target. The minimum jerk theory [38] is used to plan the motion trajectory. When jerk movement is minimized, higher-order polynomials will be reduced to fifth-order polynomials. The reference trajectory can be expressed as:

(37) $$r\left(t\right)=l_{min}+\left(l_{max}-l_{min}\right)\times \left[10{\left(\frac{t}{T}\right)}^{3}-15{\left(\frac{t}{T}\right)}^{4}+6{\left(\frac{t}{T}\right)}^{5}\right]$$

The relevant parameters of the control algorithm during the simulation are shown in Table 2.

**Figure 6:**
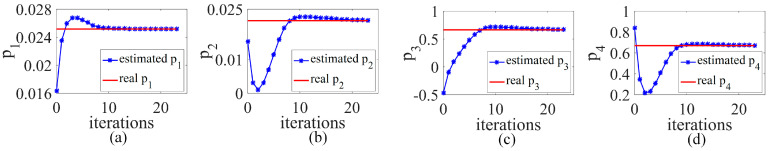
The changes of the dynamic model parameters alone with the iterative process of the control algorithm. ((**a**–**d**) represent the changes of ***P***_1_, ***P***_2_, ***P***_3_, ***P***_4_ along with iterations, respectively).

The simulation results are shown in Figure 6. We can see that using the initial model parameters obtained by the dynamic model as the starting point, the model parameters gradually converge to the real model parameters during the adaptive control learning process. This result effectively proves the convergence of the control algorithm. It also shows that the real system parameters can be obtained through the rapid iterative learning of the control algorithm and if we take the dynamic model parameters as the initial values, the trajectory tracking simulation can be achieved with better performance.

**Figure 7:**
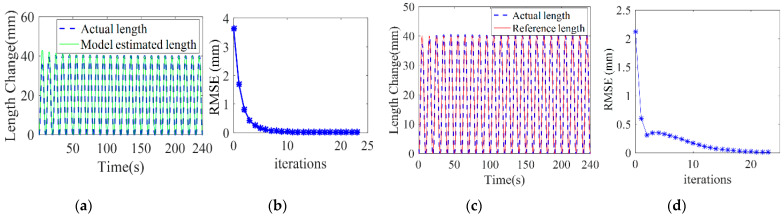
Chamber length change trajectory following simulation results. (**a**,**b**) show the comparison between the model estimated chamber length and the real chamber length. (**c**,**d**) show the comparison between the real chamber length and the reference chamber length.

In addition, as the number of iterations increases, the input pressure is brought into the real model and the estimated model, respectively. It can be seen from Figure 7a that the chamber length of the estimated model gradually tends to the real chamber length after model parameter iterative learning. The root mean square error (RMSE) between the estimated chamber length and the real length is shown in Figure 7b. After 15 iterations, the RMSE approaches 0. This result indicates that the proposed control algorithm can effectively improve the accuracy of the model parameters. In Figure 7c we can see that the real chamber length gradually tends to the reference length. The RMSE between them is presented in Figure 7d which shows that, after 20 iterations, the RMSE is 0.57 mm. Compared to the 150 mm long manipulator, this RMSE value is very small. This result indicates the good convergence performance of the control algorithm in length control.

### 5.2. Comparison with Traditional Model-Free Algorithms

**Figure 8:**
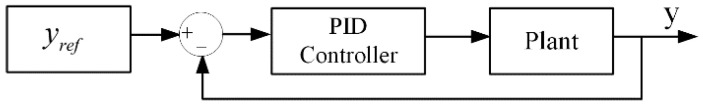
Traditional model-free PID feedback control diagram.

A diagram of a traditional model-free closed-loop feedback control algorithm is shown in Figure 8 (classic PID controller is selected here). Assume that the rest of the simulation parameters are the same as Table 2 and reference trajectory is also set as Equation (37).

**Figure 9:**
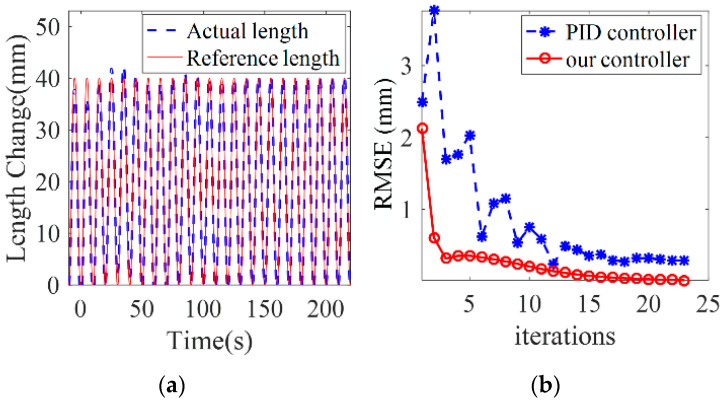
PID control simulation results. (**a**) shows the true chamber length, which can also tend to the reference length after more iterations. (**b**) the RMSE between the PID control algorithm and the proposed control algorithm.

The simulation results are shown in Figure 9. It can be seen from Figure 9a that in the first 5 control cycles, the error between the real chamber length and the reference chamber length is large. The real chamber length fluctuates greatly. RMSE is about 5 mm, which is always called the “overshoot” phenomenon. Figure 9b shows the RMSE between the reference chamber length and the real chamber length, which are obtained by the classic PID control algorithm and the proposed control algorithm, respectively. The RMSE value of the proposed parameter adaptive learning control algorithm is significantly smaller than the PID controller in the beginning. Moreover, the proposed control algorithm needs less iterations to achieve good trajectory tracking performance. After the 5th iteration of learning, the RMSE is 0.4 mm, which is lower than the RMSE value of PID method after 25 control cycles. Most importantly, the accuracy of the model parameters is gradually adjusted and perfected by the proposed control algorithm, which cannot be achieved by the traditional model-free closed-loop feedback control algorithm.

### 5.3. Effect of Initial Model Parameter Value

To analyze the influence of the initial value of the model parameters on the performance of the control algorithm, simulations withs different initial model parameters are carried out. Three different parameters are set, such as $p^{1}={\left[1;\;1;\;1;\;1\right]}^{T}$, $p^{2}={\left[-1;\;-1;\;-1;\;-1\right]}^{T}$, and $p^{3}=p_{init}$, where $p_{init}$ is the dynamic model parameter. Taking $p^{3}$ as a detailed example, all control parameters are the same as Table 2 except the initial model value.

**Figure 10:**
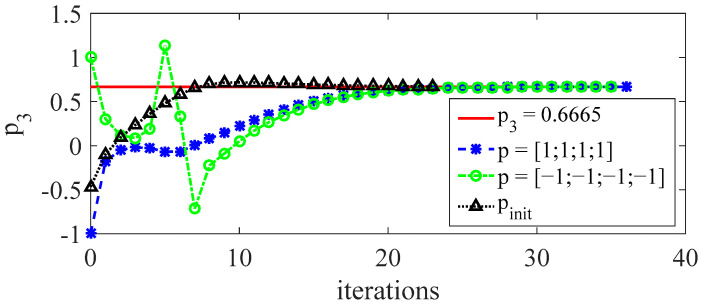
Simulation results under different initial model values. The initial values of different model parameters will converge to the real model parameters at different speeds. Taking the dynamic model parameters as the initial values can effectively reduce the number of iterations.

It can be seen from Figure 10 that for different initial values of model parameters, the algorithm can guarantee the convergence to the real model parameters. When $p_{init}$ is used as the initial model value, the convergence speed is the fastest. After the 12th iteration, it has almost converged to the real model parameters and satisfies the tolerance. However, under the other initial values, at least 20 iterations are required to meet the tolerance. This indicates that the establishment of an accurate initial values of model parameters can speed up the convergence of the control algorithm and reduce the amount of iterative learning.

### 5.4. Effect of Reference Length Change Trajectory

To analyze the influence of the reference length change trajectory, we set different types of reference trajectories to obtain the length change of the manipulator’s chamber. All control parameters are the same as Table 2, except the reference trajectories.

**Figure 11:**
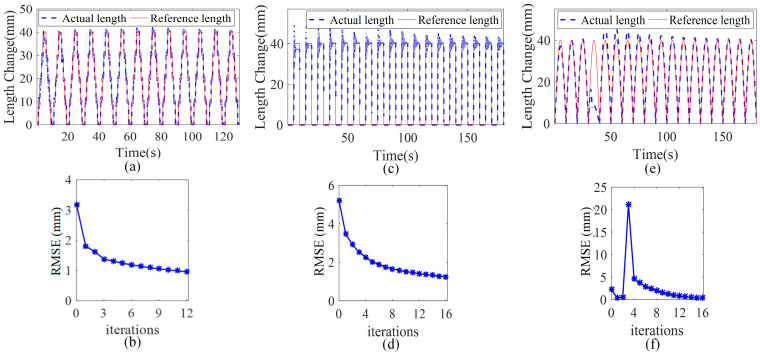
Simulation results under different reference trajectories. (**a**,**c**,**e**), show the step, rectangular wave, and sine wave as the chamber length reference, respectively; the real chamber length tends to the reference length with the number of iterations. (**b**,**d**,**f**), respectively, show the variation of the rms between the real length and the reference length under different reference trajectories.

In the simulation results in Figure 11, a trajectory tracking effect under the step length change, the rectangular wave length change, and the sine wave length change are, respectively, shown. As shown in Figure 11b,d,f, the RMSE under the sine wave reference trajectory becomes almost 0 after 15 iterations, and the RMSE under the step signal reference trajectory also changes from 5.3 mm to 1 mm after 15 iterations. Even if the step chamber length change is used as the reference trajectory, the tracking error can still gradually converge towards the decreasing direction with iterations, and the error is reduced by 72%. It is further verified that the proposed control algorithm can maintain convergence under different reference trajectories.

## 6. Experiments

**Figure 12:**
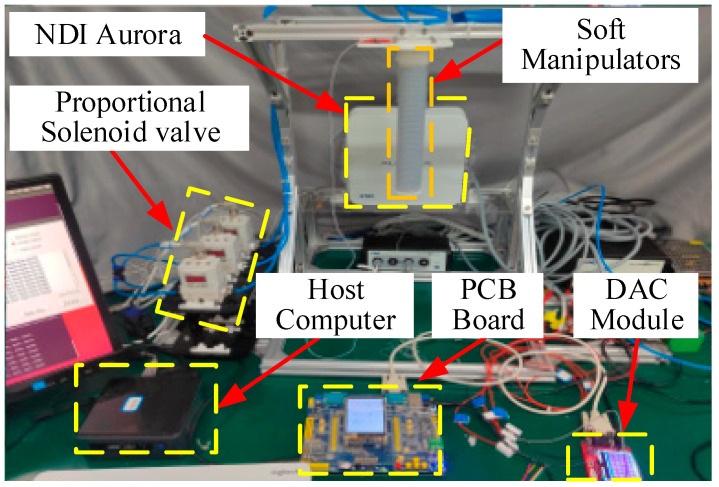
Experimental setup. The control algorithm is implemented on host computer (64-bit operating system, i5 CPU, and 16 GB RAM) based on ROS operating system. The pressure of the chamber is controlled by the SMC electric proportional valve (ITV1050). The NDI Aurora electromagnetic positioning system is used to capture the end pose of the actuator and communicate with the host through a serial port. The PCB broad is mainly used for hardware control drive.

In this section, we conduct some preliminary experiments for the single-chamber and the three-chamber soft pneumatic manipulators to verify our control method. Figure 12 shows the experimental setup of the soft manipulators. The upper computer is used to run control algorithms, dynamic model solving, and other scenarios that require higher computing power. The lower computer uses the advantages of the MCU to control and drive the proportional valve.

### 6.1. Verification Experiment of the Control Algorithm for Single-Chamber Manipulator

The single-chamber soft manipulator has only one degree of freedom. The change of the manipulator’s length is planned. The corresponding expressions for setting the desired length change trajectory are the same as Equation (34). Set the sampling period ***T*** = 8 s, and the sampling time ***T**_s_* = 0.2 s. The setting of the control algorithm parameters is consistent with the simulation. Take the single-chamber soft manipulator dynamic model parameter $p_{init}={\left[0.016\;0.015\;-0.0468\;0.837\right]}^{T}$ as the initial value of the model for iterative learning control.

**Figure 13:**
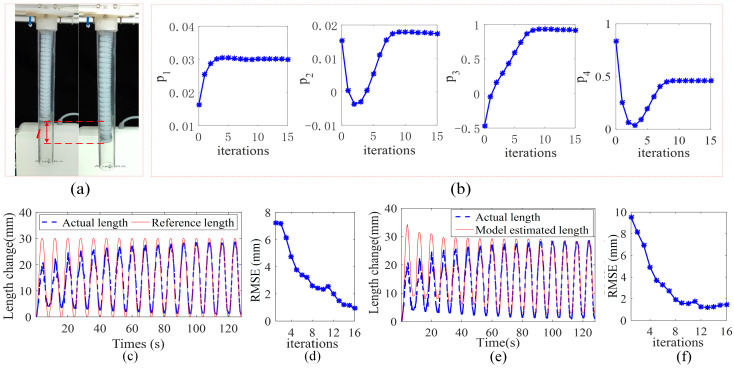
Single-chamber soft manipulator experimental results. (**a**) Schematic diagram of the experimental process. (**b**) Model parameters converge to stable values during the learning iterations. (**c**,**d**) demonstrated trajectory tracking performance. (**e**,**f**) demonstrated model parameter learning performance.

Experiment results are shown in Figure 13. As shown in Figure 13a, the manipulator begins to elongate after being pressurized. It can be seen from Figure 13c that in the first sampling period, the maximum chamber length change is 21.3 mm, which is 8.7 mm away from the expected length change peak 30 mm. The relative error is. As the number of iterations of the control algorithm increases, the real chamber length change keeps approaching the desired length change trajectory. After 10 iterations, the maximum length of the chamber change is 28.3 mm, which is 1.7 mm away from the expected peak trajectory, and the error is reduced to 5%. Figure 13d shows the RMSE of the real chamber length and the expected trajectory at each sampling time. It can be seen that RMSE has been decreasing from the initial 7.3 mm, and when the sampling ends after 16 iterations, it becomes 0.9 mm, which verifies the expected trajectory tracking performance of the proposed control algorithm. Similarly, Figure 13e,f show the performance of the control algorithm model parameter learning. The model estimated chamber length continues to tend to the actual chamber length, and the corresponding RMSE continues to decrease.

### 6.2. Verification Experiment of the Control Algorithm for Multi-Chamber Manipulator

**Figure 14:**
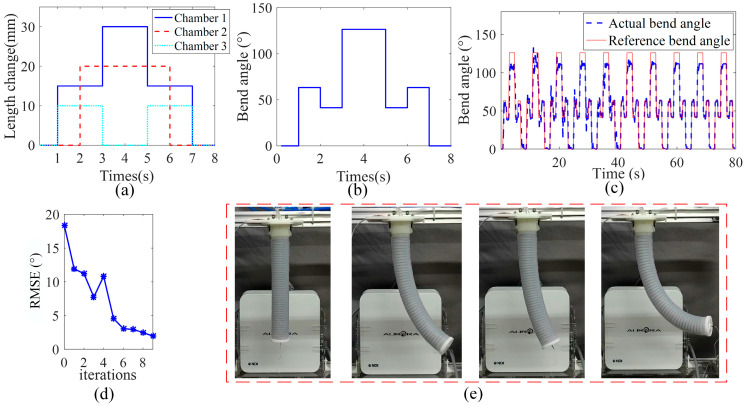
Three-chamber soft manipulator experimental results. (**a**,**b**) Set step signal as reference trajectory. (**c**) Schematic diagram of the experimental process. (**d**,**e**) shows the change process of the real bending angle and the reference bending angle, the RMSE gradually decreases.

Compared with the single-chamber actuator, the three-channel soft manipulator can perform linear contraction and bending motions in the space. We plan the trajectory of the three chamber lengths in joint space. As shown in Figure 14a, each chamber uses a different form of step signal as the desired length change trajectory, and the peak change length of each chamber is 30 mm, 20 mm, and 10 mm, respectively. For the convenience of display, the expected length change of each chamber is brought into Equation (2) to obtain the expected bending angle of the soft arm under the assumption of equal curvature segments.

The experimental results are shown in Figure 14. It can be seen from Figure 14c that in the first iteration when the dynamic model is used as the initial value of the soft arm model, the peak bending angle is 102°. Compared with the expected trajectory peak bending angle of 126°, the error between the real bend angle and the desired bend angle (obtained through desired chamber lengths) is 20.1%. As shown in Figure 14d, the RMSE of the first iteration in the multi-chamber manipulator is 18.97°. With the iterative calculation of the control program, the error gradually decreases.

This result shows that the three-chamber soft manipulator is complicated, which makes it difficult to model the dynamics model and the accuracy is low. However, with continuous iterative operation, under the effect of the parameter adaptive learning control algorithm, the actual bending angle is basically the same as the expected trajectory trend, and the RMSE in each sampling period is gradually reduced. The RMSE value of the dynamics model changes from 18.97° to 1.92° after 10 iterations. It is reduced by 87% from the original value. This result verified the effectiveness of the model-based parameter adaptive learning control algorithm for the three-chamber soft manipulator.

### 6.3. Trajectory Tracking Experiment for the Multi-Chamber Manipulator

In this experiment, trajectory planning will be carried out in the task space, and the expected trajectory length of each chamber in the joint space will be calculated through the inverse kinematics solution. The experiment is carried out in the x-y plane to plan the equilateral triangle trajectory and the side length is 125 mm.

**Figure 15:**
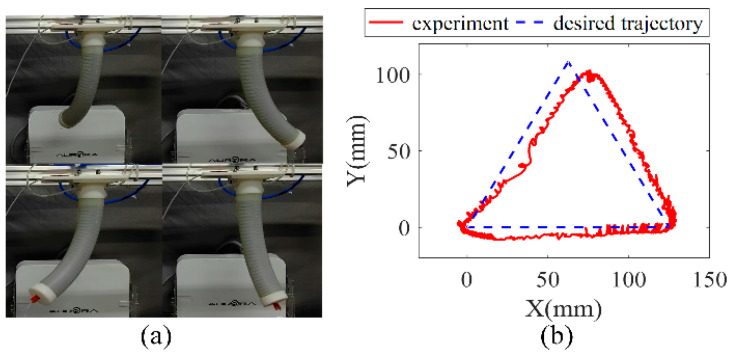
Trajectory planning experimental results. (**a**) experiment procedure. (**b**) the coordinates of the end position of the data collected by the experiment and the expected trajectory.

The experiment process is shown in Figure 15a. The end position is recorded by the NDI electromagnetic positioning system. It can be seen in Figure 15b that the experimental trajectory of the three-chamber continuous soft manipulators basically matches the desired equilateral triangle trajectory, the maximum absolute error is within 15 mm, and the average error is 4.7 mm, compared to the 150-mm-long manipulator. This result indicates that the proposed control method has good control accuracy of the soft pneumatic manipulator.

### 6.4. Discussion

From the above experiments we can see that the proposed hybrid controller has good performance in controlling the length change, bend angle, and tip end position of soft pneumatic manipulators. However, the proposed controller needs to combine the MPC and ILC together, which is more complicated than most used model-free controller or model-based controller. In this paper, the proposed method is only verified on a single-section pneumatic manipulator, its effect on a multi-section manipulator is unknown.

## 7. Conclusions

In this paper, the structural design and manufacturing process of a soft pneumatic manipulator is presented and a corresponding dynamic model based on modal method is derived which solves the problem of instability caused by the singular points in the CP kinematics. Moreover, to control the manipulator in real time and with good accuracy, a hybrid controller integrating MPC and ILC is proposed and verified through simulations and experiments. The simulation results show that the more accurate initial values of model parameters obtained by the dynamic model, the faster the convergence of the control algorithm. The experimental results show that the proposed hybrid control algorithm has good performance in controlling the manipulators’ length. During the chamber length control experiment, the RMSE value of the dynamics model changes from 18.97° to 1.92° after 10 iterations, which is an 87% reduction from the original value. In the equilateral triangle trajectory experiment, the maximum tracking error is 15 mm, and the average tracking error is 4.7 mm. This result indicates that the proposed control method has good control accuracy of the soft pneumatic manipulator. To sum up, the proposed dynamic model can be used when there exist kinematics singular solution problems, and the hybrid controller can be used when the convergence of the control algorithm is low.

Although the proposed dynamic model and the hybrid controller have been verified through experiments, the experiments are relatively simple and the prototype is a single-section manipulator. In the future, the application of the proposed method on the control of the soft multi-section manipulator will be verified and more complicated experiments will be conducted.

## Figures and Tables

**Table 1:** Dynamical Model Parameters.

$\mathrm{Chamber}\;\mathrm{Radius}\;r_{0}$	**Robot Mass** *m*	**Chamber Length** *L*_0_	$\mathrm{Chamber}\;\mathrm{Elastic}\;\mathrm{Stiffness}\;K_{e}$	$\mathrm{Chamber}\;\mathrm{Bending}\;\mathrm{Stiffness}\;K_{b}$
0.006	0.03	0.15	400 N/m	0.1 N/rad

**Table 2:** Simulation Control Parameters.

Min chamber length $l_{\min}$	0 m	Feedforward input coefficient λ	10
Max chamber length $l_{\max}$	0.04 m	Sampling time $T_{s}$	0.1 s
Output weight factor *Q*	100	Total time T	10 s
Input weight factor *R*	1	Error tolerance $\varepsilon_{t}$	0.001
Prediction horizon *H*	10	Error tolerance $\varepsilon_{r}$	0.001

## Data Availability

Not applicable.

## Appendix A

$${\left[R^{c}\right]}_{11}=\cos\left(\xi \phi \right)\cos^{2}\theta +\sin^{2}\theta$$

$${\left[R^{c}\right]}_{12}=\cos\theta \sin\theta \left(\cos\left(\xi \phi \right)-1\right)$$

$${\left[R^{c}\right]}_{13}=\sin\left(\xi \phi \right)\cos\theta$$

$${\left[R_{c}\right]}_{21}={\left[R_{c}\right]}_{12}$$

$${\left[R_{c}\right]}_{22}=\cos\left(\xi \phi \right)\sin^{2}\theta +\cos^{2}\theta$$

$${\left[R_{c}\right]}_{23}=\sin\left(\xi \phi \right)\sin\theta$$

$${\left[R_{c}\right]}_{31}=-{\left[R_{c}\right]}_{13}$$

$${\left[R_{c}\right]}_{32}=-{\left[R_{c}\right]}_{23}$$

$${\left[R_{c}\right]}_{33}=\cos\left(\xi \phi \right)$$

$${\left[P_{c}\right]}_{1}=\lambda \cos\theta \left(1-\cos\left(\xi \phi \right)\right)$$

$${\left[P_{c}\right]}_{2}=\lambda \sin\theta \left(1-\cos\left(\xi \phi \right)\right)$$

$${\left[P_{c}\right]}_{3}=\lambda \sin\left(\xi \phi \right)$$

All elements are modalized after expansion of the 6th-order multivariate Taylor series for joint space variables $\left\{l_{1},l_{2},l_{3}\right\}$ at the zero point, and the results are as follows:

$${\left[R\right]}_{11}={\left(l_{2}-2l_{1}+l_{3}\right)}^{2}\left(\frac{-\xi^{2}}{18R^{2}}+\frac{u^{2}\xi^{4}}{486R^{4}}-\frac{u^{4}\xi^{6}}{32805R^{6}}\right)+1$$

$${\left[R\right]}_{12}=-\sqrt{3}\left(l_{2}-l_{3}\right)\left(l_{2}-2l_{1}+l_{3}\right)\left[\frac{-\xi^{2}}{18R^{2}}+\frac{u^{2}\xi^{4}}{486R^{4}}-\frac{u^{4}\xi^{6}}{32805R^{6}}\right]$$

$${\left[R\right]}_{13}=\left(l_{2}-2l_{1}+l_{3}\right)\left[\frac{\xi }{3R}-\frac{2u^{2}\xi^{3}}{81R^{3}}+\frac{2u^{4}\xi^{5}}{3645R^{5}}\right]$$

$${\left[R\right]}_{21}={\left[R\right]}_{12}=-\sqrt{3}\left(l_{2}-l_{3}\right)\left(l_{2}-2l_{1}+l_{3}\right)\left[\frac{-\xi^{2}}{18R^{2}}+\frac{u^{2}\xi^{4}}{486R^{4}}-\frac{u^{4}\xi^{6}}{32805R^{6}}\right]$$

$${\left[R\right]}_{23}=-\sqrt{3}\left(l_{2}-l_{3}\right)\left[\frac{\xi }{3R}-\frac{2u^{2}\xi^{3}}{81R^{3}}+\frac{2u^{4}\xi^{5}}{3645R^{5}}\right]$$

$${\left[R\right]}_{31}=-{\left[R\right]}_{13}=-\left(l_{2}-2l_{1}+l_{3}\right)\left[\frac{\xi }{3R}-\frac{2u^{2}\xi^{3}}{81R^{3}}+\frac{2u^{4}\xi^{5}}{3645R^{5}}\right]$$

$${\left[R\right]}_{32}=-{\left[R\right]}_{23}=\sqrt{3}\left(l_{2}-l_{3}\right)\left[\frac{\xi }{3R}-\frac{2u^{2}\xi^{3}}{81R^{3}}+\frac{2u^{4}\xi^{5}}{3645R^{5}}\right]$$

$${\left[R\right]}_{33}=1-\frac{2u^{2}\xi^{2}}{9R^{2}}+\frac{2u^{4}\xi^{4}}{243R^{4}}$$

$${\left[P\right]}_{1}=-\left(3L_{0}+l_{1}+l_{2}+l_{3}\right)\left(l_{2}-2l_{1}+l_{3}\right)\left[\frac{-\xi^{2}}{18R}+\frac{u^{2}\xi^{4}}{486R^{3}}-\frac{u^{4}\xi^{6}}{32805R^{5}}\right]$$

$${\left[P\right]}_{2}=\sqrt{3}\left(3L_{0}+l_{1}+l_{2}+l_{3}\right)\left(l_{2}-l_{3}\right)\left[\frac{-\xi^{2}}{18R}+\frac{u^{2}\xi^{4}}{486R^{3}}-\frac{u^{4}\xi^{6}}{32805R^{5}}\right]$$

$${\left[P\right]}_{3}=\left(3L_{0}+l_{1}+l_{2}+l_{3}\right)\left[\frac{\xi }{3}-\frac{2u^{2}\xi^{3}}{81R^{2}}+\frac{2u^{4}\xi^{5}}{3645R^{4}}\right]$$
